# Structure and Energetics of Allosteric Regulation of HCN2 Ion Channels by Cyclic Nucleotides*[Fn FN2]

**DOI:** 10.1074/jbc.M115.696450

**Published:** 2015-11-11

**Authors:** Hannah A. DeBerg, Peter S. Brzovic, Galen E. Flynn, William N. Zagotta, Stefan Stoll

**Affiliations:** From the Departments of ‡ Chemistry,; § Physiology and Biophysics, and; ¶ Biochemistry, University of Washington, Seattle, Washington 98195

**Keywords:** allosteric regulation, cyclic nucleotide, electron paramagnetic resonance (EPR), electrophysiology, fluorescence anisotropy, ion channel, nuclear magnetic resonance (NMR), double electron-electron resonance (DEER)

## Abstract

Hyperpolarization-activated cyclic nucleotide-gated (HCN) ion channels play an important role in regulating electrical activity in the heart and brain. They are gated by the binding of cyclic nucleotides to a conserved, intracellular cyclic nucleotide-binding domain (CNBD), which is connected to the channel pore by a C-linker region. Binding of cyclic nucleotides increases the rate and extent of channel activation and shifts it to less hyperpolarized voltages. We probed the allosteric mechanism of different cyclic nucleotides on the CNBD and on channel gating. Electrophysiology experiments showed that cAMP, cGMP, and cCMP were effective agonists of the channel and produced similar increases in the extent of channel activation. In contrast, electron paramagnetic resonance (EPR) and nuclear magnetic resonance (NMR) on the isolated CNBD indicated that the induced conformational changes and the degrees of stabilization of the active conformation differed for the three cyclic nucleotides. We explain these results with a model where different allosteric mechanisms in the CNBD all converge to have the same effect on the C-linker and render all three cyclic nucleotides similarly potent activators of the channel.

## Introduction

The ability of proteins to dynamically sample different conformational states is critical to their function. Therefore, understanding the conformational landscape is crucial for understanding how biological function arises from the structure of proteins. In the case of ion channel proteins, allosteric regulation of channel opening involves a number of distinct conformational states. Channels are regulated by a variety of stimuli, including voltage, temperature, membrane stretch, and ligand binding (1, 2).

**FIGURE 1.**
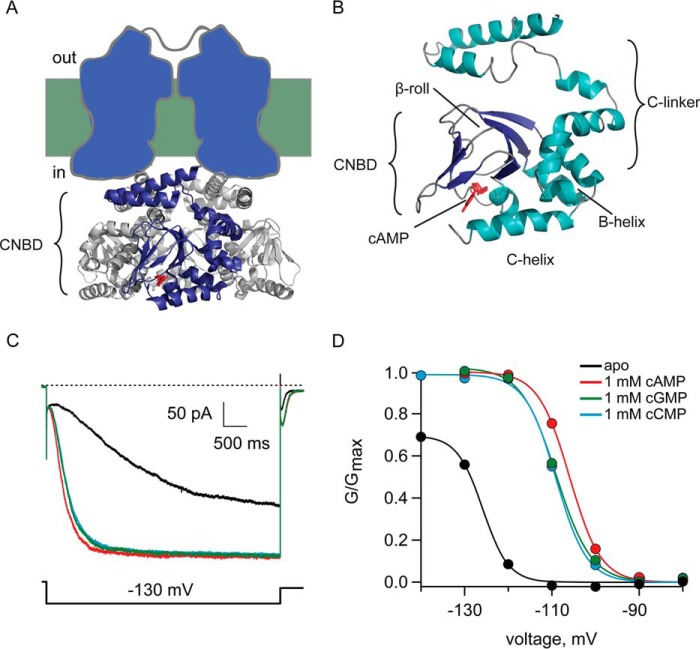
**Cyclic nucleotide regulation of HCN2 channels.** *A*, schematic structure of the channel that shows a tetrameric CNBD and C-linker (Protein Data Bank ID 3ETQ) with cAMP bound in *red. B*, crystal structure of the C-linker and CNBD, with cAMP bound. *C*, representative current traces elicited by a −130 mV hyperpolarizing step applied to channels expressed in oocytes in the inside-out patch-clamp configuration. Currents were recorded in the absence of ligand (*black*), 1 mm cAMP (*red*), cGMP (*green*), or cCMP (*cyan*). *D*, normalized conductance-voltage relationships of HCN2 channels from the patch shown in *C*.

In the hyperpolarization-activated cyclic nucleotide-gated (HCN)^4^ ion channel, the binding of cyclic nucleotide ligands produces conformational rearrangements that stabilize its open state (3–5). HCN channels play important roles in the heart, where they regulate pacemaking activity, and in the brain, where they control the resting membrane potential and modulate dendritic integration (4). Among members of the voltage-gated potassium channel superfamily, HCN channels have two features that render them unique (3). First, unlike most members of their family that open in response to membrane depolarization, HCN channels open in response to membrane hyperpolarization. Second, they are regulated by the binding of cyclic nucleotides to a conserved, intracellular cyclic nucleotide-binding domain (CNBD, Fig. 1*A*).

In the CNBD, cyclic nucleotides bind between the β-roll, a subdomain consisting of eight anti-parallel β-strands, and the C-helix (Fig. 1*B*). Previous structural studies have revealed that the C-helix moves in toward the β-roll upon cAMP binding (6–10). A domain called the C-linker connects the CNBD to the transmembrane domains. The C-linker is the site of nearly all of the inter-subunit interactions in the carboxyl-terminal region of the tetrameric channel. There is evidence in both HCN and CNG (cyclic nucleotide-gated) channels that rearrangement of the C-linker plays a key role in coupling cyclic nucleotide-induced conformational changes in the CNBD to pore opening (11–16). Cyclic nucleotide binding to the CNBD increases the rate and extent of channel activation and shifts it to less hyperpolarized voltages (3, 4). cAMP is the physiological agonist of the HCN2 channel isoform that is expressed in the sinoatrial node, dorsal root ganglia, and basal ganglia (3). 3′,5′-Cyclic guanosine monophosphate (cGMP) and 3′,5′-cyclic cytidine monophosphate (cCMP) also bind to the CNBD and regulate the channel (6, 9, 17). Previously, it has been reported that cGMP is a full agonist, producing a depolarizing shift in activation voltage similar to cAMP, but with a higher EC_50_ (6, 9). In contrast, cCMP has been reported to act as a partial agonist, producing only 60% of the depolarizing shift in activation voltage produced by cAMP at saturating concentrations (17).

In this study, we investigated the mechanism of allosteric regulation of HCN2 by cAMP, cGMP, and cCMP. We examined conformational changes of the isolated CNBD that accompany cyclic nucleotide binding with biophysical and biochemical approaches. Nuclear magnetic resonance (NMR) and electron paramagnetic resonance (EPR) data provided residue-level information on conformational changes resulting from cyclic nucleotide binding. We found differences in conformation of the isolated CNBD depending on which cyclic nucleotide was bound. We also found differences in the extent to which different cyclic nucleotides stabilized the activated conformation of the CNBD. Despite the differential effects of the ligands on the isolated CNBD, all three cyclic nucleotide agonists produced similar effects on pore opening in the intact channel. To address how differences in the CNBD conformation can result in similar gating effects, we analyzed the energetics associated with ligand binding to the CNBD and compared it to the energetics for the intact channel. Our analysis supports a model where differential allosteric mechanisms in the CNBD all converge to have the same effect on the C-linker and make all three cyclic nucleotides similarly potent channel activators.

## Experimental Procedures

### 

#### 

##### Molecular Biology

For NMR experiments, cDNA encoding residues 488–640 of murine HCN2 in a cysteine-free background (HCN2-CNBD) was cloned into the pETM11 vector (10). The vector contains an N-terminal poly-His tag separated from the channel gene by a TEV (tobacco etch virus)-cleavable linker. For double electron-electron resonance (DEER) experiments, the gene encoding residues 443–640 of a cysteine-free fragment of the murine HCN2 ion channel (HCN2-CL +CNBD) was cloned into the pMALc2T vector (New England Biolabs) (10). The vector contains an N-terminal maltose-binding protein tag separated from the channel gene by a thrombin-cleavable linker. For EPR studies, cysteine mutations were introduced using standard PCR-based techniques. All constructs were confirmed with fluorescence-based automated sequencing. The cDNA encoding the full-length murine HCN2 channel in the pGEMHE vector was kindly provided by Steven Siegelbaum and Bina Santoro (Columbia University).

##### Electrophysiology

Electrophysiology experiments were performed on full-length murine HCN2 channels. cRNA for HCN2 channels was transcribed using the mMessage Machine T7 transcription kit (Ambion) and expressed in defolliculated *Xenopus laevis* oocytes as previously described (18). The vitelline membranes were manually removed, and currents were recorded with an EPC-10 patch clamp amplifier (HEKA Elektronik) in the inside-out patch clamp configuration (19). Patch pipettes were pulled from borosilicate glass (VWR) and fire polished to a resistance of 0.3–0.6 MΩ. Both pipette and bath solutions contained 130 mm KCl, 3 mm HEPES, 0.2 mm EDTA, adjusted to pH 7.2 with KOH. Solutions containing 1 mm cyclic nucleotides were perfused onto the patches as indicated using a RSC-100 solution changer (BioLogic).

Currents were elicited by a series of incremental test pulse voltages (5 s) in the range of −90 to −140 mV followed by a voltage step to −40 mV (500 ms). The voltage stimulus protocol was applied every 10 s from a holding potential of 0 mV. Peak tail current amplitudes were measured from the −40 mV voltage step and plotted *versus* the test pulse voltage. The leak conductance was corrected by subtracting the voltage-independent conductance at depolarized voltages. Currents were normalized to the peak tail current measured in the presence of cAMP, plotted against the test voltages, and fit with the Boltzmann equation,

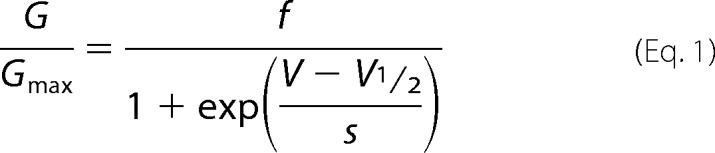
 where *G*_max_ is the maximum conductance measured for the patch in the presence of cAMP, *f* is the fraction of *G*_max_ at maximally hyperpolarizing *V*, *V* is the test pulse voltage, *V*_½_ is the half-activation voltage, and *s* is the slope factor. All fittings were performed with IGOR Pro (Wavemetrics).

##### Protein Expression, Purification, and Spin Labeling

The HCN2-CL + CNBD and HCN2-CNBD constructs were transfected into BL21(DE3) *Escherichia coli* cells, and 2–4-liter cultures of cells were grown at 37 °C. For NMR experiments, the cells were grown in MOPS minimal media, supplemented with ^15^NH_4_Cl (1 g/liter). For all other experiments, the cells were grown in Luria Broth. At an optical density of 0.6–0.8, the cells were induced with 1 mm isopropyl β-d-1-thiogalactopyranoside and grown overnight at 18 °C. Cells were pelleted by centrifugation at 4,000 × *g* at 4 °C for 10 min and resuspended in 150 mm KCl and 30 mm HEPES, pH 7.2. DNase at a final concentration of 5 μg/ml and two tablets of protease inhibitors (Roche, cOmplete EDTA-free) were added to the buffer. The resuspended cells were lysed by an Emulsiflex-C3 homogenizer (Avestin) and clarified by centrifugation at 186,000 × *g* at 4 °C for 45 min.

For NMR experiments with HCN2-CNBD, the lysate was then purified on a Ni^2+^ affinity resin column (HisTrap HP, GE Healthcare). The His_8_ tag was removed by TEV protease cleavage overnight at 4 °C. To remove the cleaved tag, the sample was purified by ion exchange chromatography. The sample was diluted in buffer containing 10 mm KCl, 30 mm HEPES, 10% glycerol, pH 7.2, loaded on an SP-Sepharose column (GE, HiTrap SP FF), and eluted with a continuous gradient between 10 mm and 1 m KCl. Fractions enriched in protein were concentrated and further purified over a size exclusion column (GE Healthcare, HiLoad 16/600 Superdex 200). Fractions containing protein were pooled and concentrated to ∼300 μm using a 10-kDa MWCO centrifugal filter (GE, Vivaspin).

For experiments with HCN2-CL + CNBD, the bacterial lysate was purified with amylose affinity chromatography, and maltose-binding protein was cleaved off by thrombin incubation at room temperature for 4 h. The protein (10–50 μm) was then spin-labeled with 100 μm S-(1-oxyl-2,2,5,5-tetramethyl-2,5-dihydro-1*H*-pyrrol-3-yl)methyl methanesulfonothioate (Toronto Research Chemicals) per cysteine mutation for 1 h at room temperature for EPR experiments. To remove maltose-binding protein and excess spin label, the sample was purified by ion exchange chromatography. It was diluted in buffer containing 10 mm KCl, 30 mm HEPES, 10% glycerol, pH 7.2, and purified on an SP-Sepharose column (GE Healthcare, HiTrap SP FF). Fractions containing protein were pooled and concentrated to 50–200 μm using a 10-kDa MWCO centrifugal filter (GE, Vivaspin). Cyclic nucleotides were added at the concentrations indicated in the text and figures.

##### Fluorescence Anisotropy

Fluorescence anisotropies were recorded using a Fluorolog 3 spectrophotometer and FluorEssence software (HORIBA Jobin Yvon). To measure the affinity of HCN2-CL + CNBD for the fluorescent ligand, 8-Fluo-cAMP (BIOLOG), the anisotropy of 10 nm 8-Fluo-cAMP with varying concentrations (0–10 μm) of HCN2-CL + CNBD was recorded. Measurements were repeated in triplicate and the mean is plotted with bars reporting the standard error. To calculate the binding affinity, the plot of fluorescence anisotropy *versus* total protein concentration was fit with,

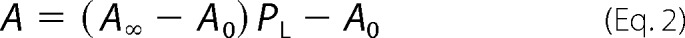
 where *A*_∞_ and *A*_0_ are the fluorescence anisotropies at saturating concentration of HCN2 and in the absence of HCN2-CL + CNBD, respectively. *P*_L_ is the fraction of 8-Fluo-cAMP bound to HCN2-CL + CNBD (20) and is given by,

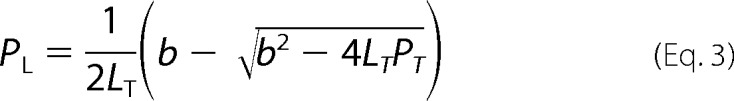
 with *b* = *P*_T_ + *L*_T_ + *K*_D_. *P*_T_ is the total concentration of HCN2-CL + CNBD, *L*_T_ is the total concentration of 8-Fluo-cAMP, and *K*_D_ is the dissociation constant. Fits of these equations to the data were performed in Matlab.

To determine the affinity of HCN2-CL + CNBD for non-fluorescent cyclic nucleotides, competition experiments were performed. The HCN2-CL + CNBD concentration was fixed at 2 μm, with 10 nm 8-Fluo-cAMP, and fluorescence anisotropy was recorded in the presence of varying concentrations (0.5–10 mm) of non-fluorescent cAMP, cCMP, or cGMP. Data were scaled to fall into the range of 0–1. Assuming competitive binding of 8-Fluo-cAMP (denoted as *L*) and the non-fluorescent cyclic nucleotide (denoted as *M*) to HCN2-CL + CNBD (denoted as *P*), the system can be described by the following equilibria: *P* + *L* ⇌ *PL*, *P* + *M* ⇌ *PM* with the associated dissociation constants,

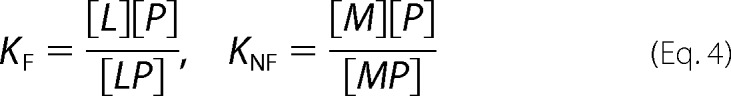
 for 8-Fluo-cAMP and a non-fluorescent cyclic nucleotide, respectively. For this situation, the scaled anisotropy *A*_s_ (scaled to the range 0–1) is described by Ref. 21,

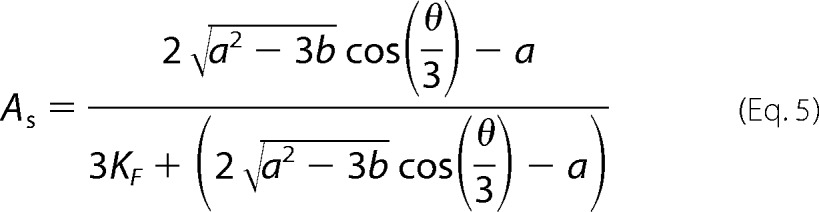
 with *a*, *b*, and θ defined as follows.

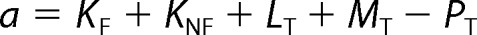


##### NMR

NMR spectra were recorded on a 600 MHZ Bruker Avance III spectrometer equipped with a cryoprobe. All spectra were recorded at 25 °C in 30 mm HEPES, 150 mm KCl, 10% D_2_O, pH 7.2. ^1^H,^15^N-TROSY spectra were collected on a series of samples containing a constant concentration (100 μm) of ^15^N-labeled HCN2-CNBD in the presence of cyclic nucleotides (0, 25, 50, 100, and 200 μm). An additional spectrum was collected at 500 μm for cCMP only. NMR data were processed using NMRPipe and visualized using NMRView (22, 23). Chemical shift perturbations were calculated according to the average scaled Euclidean distance moved,

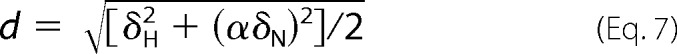
 where δ_H_ is the ^1^H chemical shift change and δ_N_ is the ^15^N chemical shift change (24). The scaling factor α = 0.15 was selected as the ratio of the spectral width in the ^1^H dimension (3.5 ppm) to the spectral width in the ^15^N dimension (23 ppm).

##### DEER Experiments

For DEER experiments, the protein was buffer exchanged into D_2_O with 150 mm KCl, 30 mm HEPES, and 10% glycerol using a PD-10 column (GE Healthcare). It was diluted to 50 μm and cyclic nucleotides were added at 1–5 mm. Approximately 30 μl of each protein and cyclic nucleotide sample was inserted into a 1.65-mm outer diameter quartz tube (Sutter, Q165-115-10) and flash frozen in liquid nitrogen. Experiments were performed at X-band (1 kW) and Q-band (3 W) using a Bruker EleXsys E580 spectrometer. DEER data for the V537C/A624C and V537C/R635C HCN2-CL + CNBD mutants were acquired at X-band (9–10 GHz) with an overcoupled dielectric resonator (Bruker MD4). All other data were taken at Q-band (33–35 GHz) with an overcoupled dielectric resonator (Bruker EN5107D2). Experiments were performed at 60 K using a liquid helium cooling system (Oxford). The four pulse, dead-time free DEER sequence (π/2)_probe_ − τ_1_ − (π)_probe_ −(τ_1_ + *t*) − (π)_pump_ − (τ_2_ − *t*) − (π)_probe_ − τ_2_ was used with 22-ns π/2 probe pulses and 44-ns π probe and pump pulses at Q-band and 12-ns π/2 probe pulses and 24-ns π probe and pump pulses at X-band. Pulse delays were 120 ns for τ_1_ and between 1800 and 4000 ns for τ_2_. The delay *t* was varied in 10-ns increments from −60 ns to between 1800 and 4000 ns, depending on τ_2_. The pump frequency was set to the center of the resonator mode to enable the shortest possible pump pulse and the largest possible excitation bandwidth. The field was adjusted such that the spectral maximum occurred at the same frequency to maximize the number of spins being pumped. The probe frequency was set 62 MHz lower to maximize the number of probe spins, whereas avoiding excitation overlap between pump and probe pulses. An eight-step phase cycling protocol combined with averaging with a repetition time of 2 ms was used to collect data. The measurement time per sample was 10–16 h.

##### DEER Data Analysis

The rotamer predictions shown in Figs. 5 and 6 were obtained using MMM software (25). DEER distance distributions were obtained using DeerAnalysis2013 (26). A homogeneous three-dimensional background was used for background correction. Time traces were converted to distance distributions using Tikhonov regularization, a model-free least-squares approach. The regularization parameter was visually optimized separately for each data set according to the L-curve criterion. Regularization parameter values of 10 or 100 were used for all datasets. To estimate errors in the obtained distance distributions, the noise in the time domain traces was linearly transformed to the distance domain. The shaded error bands shown in the distance distributions correspond to 2 S.D. (±2σ) of the time domain noise.

## Results

### 

#### 

##### Cyclic Nucleotides Regulate HCN2 Ion Channels

To directlycompare the effects of the three nucleotides on channel gating, we performed electrophysiology experiments. Different cyclic nucleotides were applied to inside-out patches excised from *X. laevis* oocytes expressing HCN2 channels. Fig. 1*C* shows currents from a patch in response to a −130 mV step in the absence of cyclic nucleotide and in the presence of 1 mm cAMP, cGMP, or cCMP. The conductance-voltage relationships measured from the tail currents (Fig. 1*D*) of four patches were fitted with a Boltzmann equation (Equation 1), and the results are summarized in Table 1. In Fig. 1*D*, *G*_max_ indicates the maximal current for cAMP. Application of the three cyclic nucleotides resulted in similar increases in maximal current at hyperpolarized voltages relative to unbound channels. The shifts Δ*V*_½_ caused by cGMP and cCMP (15.8 ± 0.71 and 14.5 ± 1.94 mV, respectively) were slightly smaller than the shift caused by cAMP (18.3 ± 1.19 mV), indicating that cGMP and cCMP were slightly less effective agonists than cAMP. Previously, Zong *et al*. (17) reported a Δ*V*_½_ for cCMP of 60% of that of cAMP. They also reported less negative values of *V*_½_ and smaller values of Δ*V*_½_ for both cCMP and cAMP. Differences in electrophysiology conditions or expression systems are likely responsible for the discrepancies between their results and ours. Our cAMP and cGMP measurements agree with previously published values (5, 6, 9). From our data, we conclude that all three cyclic nucleotides are highly effective HCN2 agonists.

**TABLE 1:** **Boltzmann fit parameters** Data are given as the average of four patches ± S.E.

Ligand	*V*_1/2_ (mV)	*s* (mV)	*f*
Apo	−130 ± 2	3.1 ± 0.1	0.73 ± 0.02
cAMP	−112 ± 2	3.9 ± 0.2	1
cGMP	−114 ± 2	3.8 ± 0.1	0.98 ± 0.01
cCMP	−115 ± 2	3.9 ± 0.1	1.00 ± 0.01

##### Cyclic Nucleotides Have Different Affinities to the Isolated CNBD

Cyclic nucleotides regulate the channel by binding and inducing conformational changes in the CNBD that are propagated through the C-linker. These conformational changes are coupled, in turn, to pore opening (3, 4). To probe the effect of cyclic nucleotide binding to the CNBD, we used a cysteine-free carboxyl-terminal fragment of the HCN2 channel containing the C-linker and CNBD (HCN2-CL + CNBD) (Fig. 1*B*). This construct has been crystallized previously and was found to have a structure almost identical to the wild-type fragment with an α-carbon root mean square deviation of 0.6 Å (10).

**FIGURE 2.**
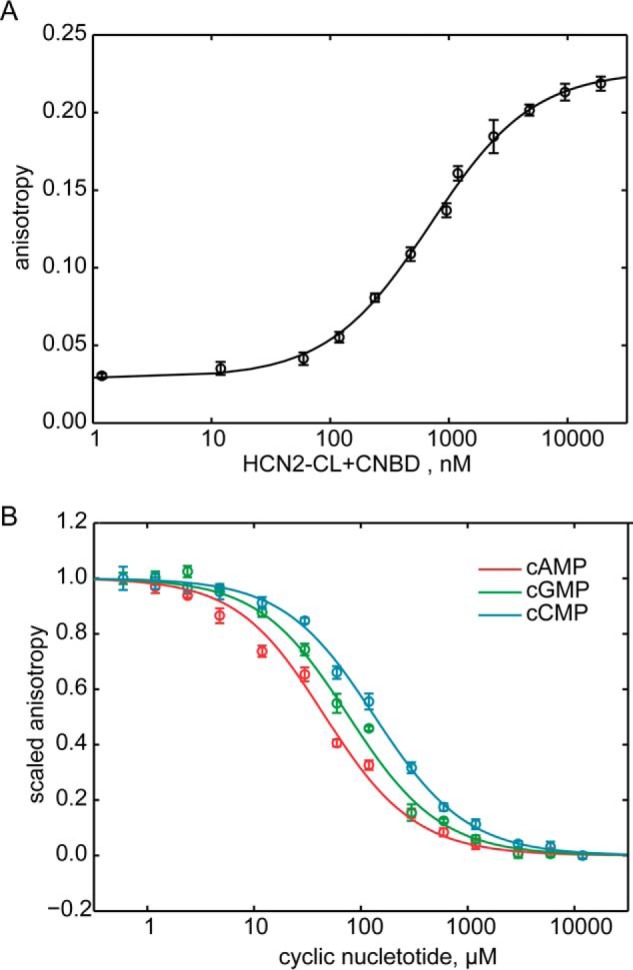
**Fluorescence anisotropy measurements of cyclic nucleotide affinity for HCN2-CL + CNBD.** *A*, fluorescence anisotropy of 10 nm 8-Fluo-cAMP as a function of total HCN2-CL + CNBD concentration. *B*, fluorescence anisotropy of 10 nm 8-Fluo-cAMP and 2 μm HCN2-CL + CNBD as a function of total non-fluorescent cyclic nucleotide concentration. Data from non-fluorescence cAMP competition are shown in *red*, cGMP in *green*, and cCMP in *cyan*.

To measure the affinity of cyclic nucleotides for HCN2-CL + CNBD, we performed fluorescence anisotropy experiments. Fluorescence anisotropy reports the tumbling rate of a fluorescent probe in solution by exciting it with polarized light and measuring the polarization of the emission. When free in solution, a small fluorescent probe will rapidly tumble and the emitted light will be depolarized compared with the excitation, resulting in low values of anisotropy. When bound to a large protein, the tumbling rate is decreased and the emission will be less depolarized (27), giving higher values of anisotropy. Fig. 2*A* shows the anisotropy of 10 nm 8-Fluo-cAMP as a function of the total HCN2-CL + CNBD concentration. Fitting the measurements with Equation 2 (see “Experimental Procedures”) resulted in an affinity for 8-Fluo-cAMP of 0.69 ± 0.04 μm. To measure the affinity of non-fluorescent cyclic nucleotides, we fixed the concentrations of 8-Fluo-cAMP and HCN2-CL + CNBD at 10 nm and 2 μm, respectively, and competed off 8-Fluo-cAMP with increasing concentrations of non-fluorescent cAMP, cGMP, or cCMP (Fig. 2*B*). The measurements were fit with Equation 4 (see “Experimental Procedures”), which yielded dissociation constants of 8 ± 1, 16 ± 2, and 26 ± 3 μm for cAMP, cGMP, and cCMP, respectively. These data are in agreement with previous affinity measurements on the isolated CNDB and indicate that the cysteine-free fragment binds cyclic nucleotides with similar affinities as previously measured for the isolated CNBD (28, 29). Like previous measurements, these affinities are less than the ≈0.5 μm cAMP and ≈1.5 μm cGMP affinities measured for intact channels (6, 17).

##### NMR Indicates Different Cyclic Nucleotide-bound States Have Different Conformations

**FIGURE 3.**
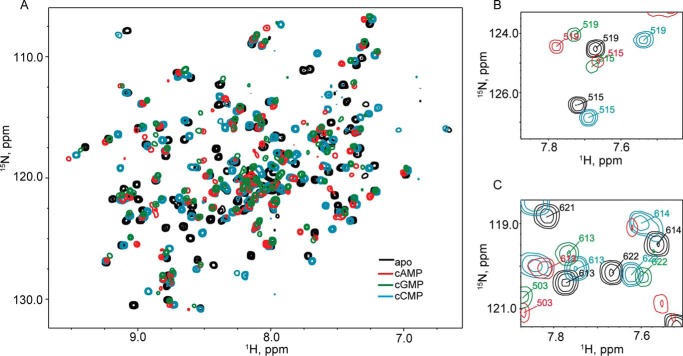
**Two-dimensional NMR spectra of HCN2-CNBD.** *A*, ^1^H,^15^N-TROSY spectrum of 100 μm HCN2-CNBD either unbound (*black*), or in the presence of 200 μm cAMP (*red*), 200 μm cGMP (*green*), and 500 μm cCMP (cyan). *B*, enlargement of *A* showing resonances of selected residues from the C-linker (515 and 519). *C*, enlargement of *A* showing resonances of selected residues from the B-helix (613 and 614) and C-helix (622).

To examine the effect of cyclic nucleotide binding at the level of individual residues, we turned to NMR spectroscopy. We used a truncated construct, HCN2-CNBD, consisting of residues 488–640 of wild-type HCN2 and labeled it with ^15^N (30). This construct lacks C-linker helices required for tetramerization, guaranteeing it will be a monomer and, hence, small enough to be suitable for solution NMR. We collected ^1^H,^15^N-TROSY spectra of 100 μm HCN2-CNBD (Fig. 3). Resonance assignments for many (62%) residues of ligand-free (apo) HCN2-CNBD could be made from assignments published by Saponaro *et al.* (8) for a very similar fragment of human HCN2. Tracking resonance shifts with increasing concentrations of cAMP, cGMP, and cCMP, we could confidently assign resonances for 33% of residues in the presence of 200 μm cAMP, 40% with 200 μm cGMP, and 50% with 500 μm cCMP. At these cyclic nucleotide concentrations, over 90% of HCN2-CNBD is expected to be ligand-bound based on our determined affinities. Although NMR was performed on a shorter construct than used in fluorescence experiments, this should result in an underestimate of occupancy because previous experiments have estimated that the affinity of this shorter construct for cyclic nucleotides is slightly higher (31).

^1^H and ^15^N chemical shifts of backbone amide groups are very sensitive to the local chemical environment and can be perturbed by the binding of a ligand and/or an allosteric conformational change (24). We observed significant chemical shift perturbations upon the addition of each ligand for residues far from the cyclic nucleotide binding site. These perturbations were not likely the result of ring current effects from the ligand (with a relatively weak distance dependence of 1/*r*^3^), but more likely the result of allosteric conformational changes caused by ligand binding (Fig. 3*A*). The observed chemical shift perturbations of these residues differed depending on which cyclic nucleotide was bound (Fig. 3, *B* and *C*). Fig. 3*B* shows the chemical shifts of residues 515 and 519 in the C-linker, and Fig. 3*C* shows residues 613 and 614 in the B-helix and residue 622 in the C-helix. In each case, the magnitudes and directions of the chemical shifts were different for the three cyclic nucleotides. We observed no overall systematic pattern, indicating that each cyclic nucleotide species had an effect that was distinct from the others.

**FIGURE 4.**
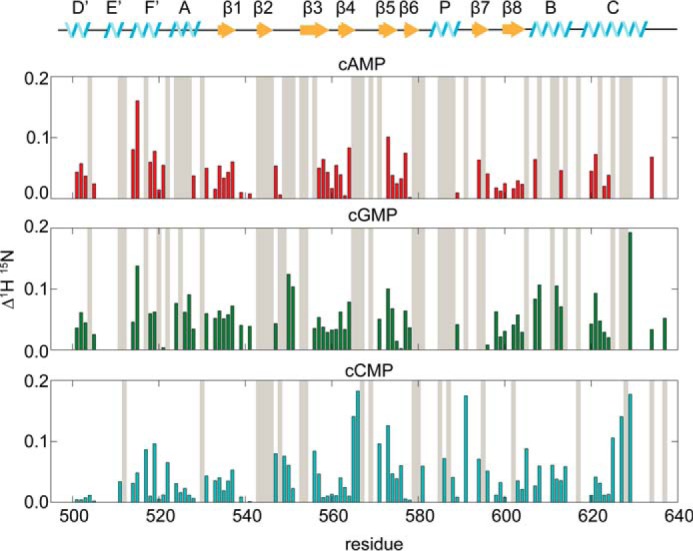
**Chemical shift perturbations of HCN2-CNBD.** Chemical shift differences between apo and 200 μm cAMP (*red*), apo and 200 μm cGMP (*green*), and apo and 500 μm cCMP (*cyan*) bound HCN2-CNBD. Resonances that could not be unambiguously assigned after titration of cyclic nucleotides are indicated by *gray shaded bars*.

The magnitudes of the chemical shift perturbations for the assigned resonances are plotted in Fig. 4. Residues with chemical shifts that disappeared or could not be tracked upon addition of cyclic nucleotide are shown in *gray*. A comparison of the chemical shift perturbations induced by binding cGMP or cCMP relative to chemical shift perturbations induced by binding cAMP is shown in supplemental Fig. S1. If the CNBD were to sample only two conformations, the chemical shift perturbations of cAMP, cGMP, and cCMP would be expected to be collinear. However, a comparison of chemical shift perturbations indicates that they are not collinear (Figs. 3, *B* and *C*, and supplemental Fig. S1). In contrast to these findings, a previous study with the HCN4 isoform found that the cCMP and cAMP chemical shift perturbations fell largely along a line (32). Differences in the HCN isoform used in these experiments may be the cause of this discrepancy. Our results indicate that the HCN2 CNBD is not limited to sampling two conformations corresponding to cyclic nucleotide bound and unbound, but rather operates in a more complex conformational landscape, adopting different conformations depending on which cyclic nucleotide species is bound. The nature of the structural differences between these conformations is unknown at this point.

##### DEER Reveals Site-specific Conformational Differences

To directly probe conformational changes associated with cyclic nucleotide binding, we performed DEER. DEER is a pulsed EPR technique that measures the magnetic dipole-dipole interaction between two spin labels attached to a protein to determine their separation (33–35). It functions in the range of 15–80 Å. A strength of DEER is that it reports distance distributions, not average separations, making it able to report multiple conformations in the sample including their relative populations. We introduced pairs of cysteines into HCN2-CL + CNBD and labeled these sites with the spin label S-(1-oxyl-2,2,5,5-tetramethyl-2,5-dihydro-1*H*-pyrrol-3-yl)methyl methanesulfonothioate. For DEER experiments, we selected pairs of residues spread across the β-roll (537, 563), B-helix (608, 616), and C-helix (624, 635) and measured distance distributions in the absence and presence of saturating (1 mm) cAMP, cGMP, and cCMP.

**FIGURE 5.**
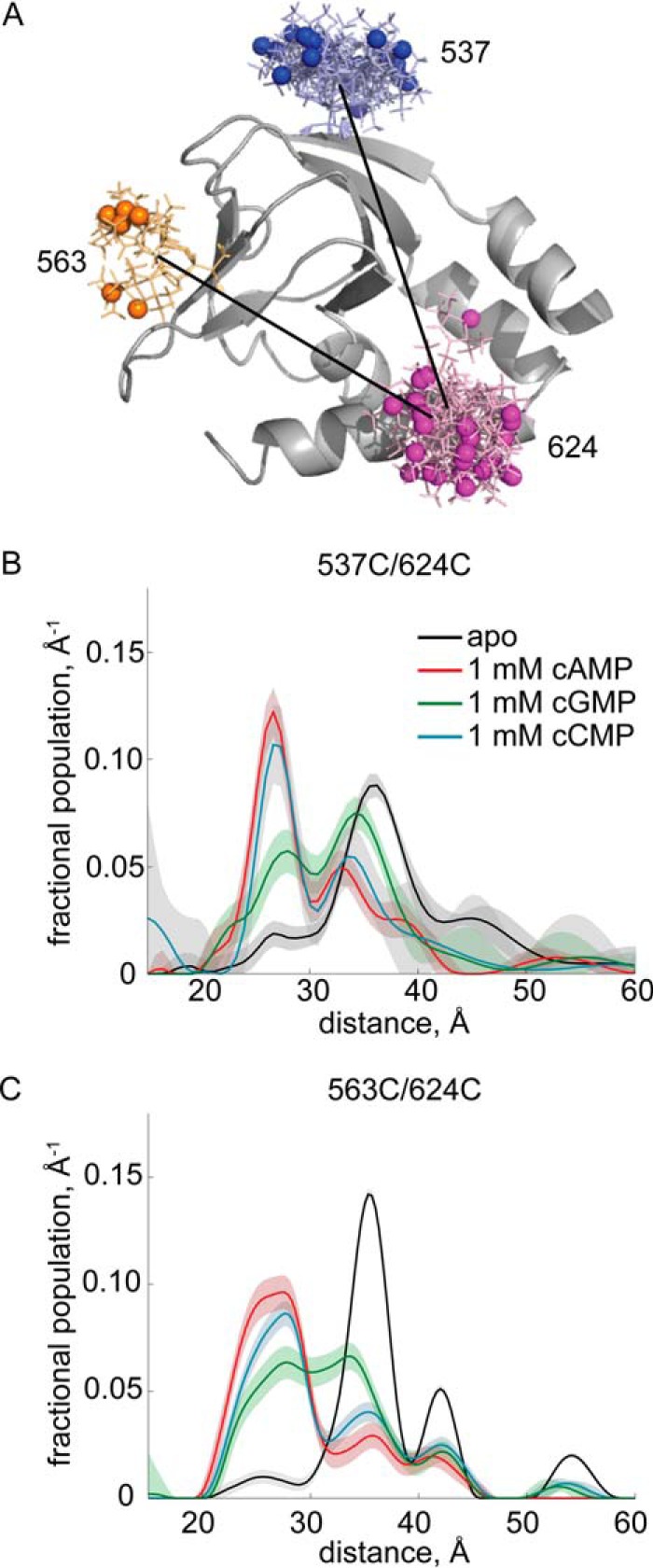
**DEER distance distributions involving residue 624.** *A*, structure of HCN2-CL + CNBD (Protein Data Bank code 3ETQ), in *gray*, with predicted spin label rotamers, in color, at residues 537, 563, and 624. Spin label rotamers were predicted using MMM software (25). *B*, DEER distance distributions of HCN2-CL + CNBD 537/624. *C,* DEER distance distributions of HCN2-CL + CNBD 563/624. Distance distributions for apo HCN2-CL + CNBD are in black, of HCN2-CL + CNBD bound to 1 mm cAMP in *red*, bound to 1 mm cGMP in *green*, and bound to 1 mm cCMP in *cyan*.

Our previous DEER experiments revealed that residue 624, on the proximal end of the C-helix, moves 9 Å toward the β-roll upon binding cAMP (7, 30). We performed DEER measurements on HCN2-CL + CNBD spin labeled at 563/624 and 537/624 and confirmed the Puljung *et al.* (7) observation that, for both positions in the β-roll, binding cAMP shifted the distance distribution by 9 Å toward shorter distances (Figs. 5 and supplemental Fig. S2). For both positions in the β-roll, we found that cCMP binding produced a distance distribution almost identical to that of cAMP (Fig. 5, *B* and *C*). cGMP binding produced a bimodal distance distribution, with one mode at shorter distances, similar to the predominant cAMP and cCMP bound modes, and a second mode at larger distances, near the dominant separation observed in the apo state. This suggests that cGMP is less able to drive the conformational change at residue 624 than cAMP or cCMP. This is surprising because, in the intact channel, cGMP is about as effective an agonist as cAMP, and, if anything, slightly more effective than cCMP.

**FIGURE 6.**
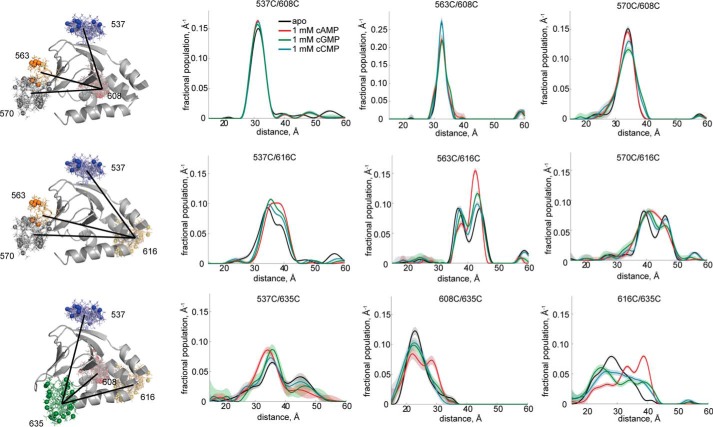
**Other DEER distance distributions.** The *top row* displays distributions involving residue 608 at the beginning of the B-helix, the *center row* displays distributions involving residue 616 at the end of the B-helix, and the *bottom row* shows distributions involving residue 635 at the end of the C-helix. Distance distributions for apo HCN2-CL + CNBD are in *black*, of HCN2-CL + CNBD bound to 1 mm cAMP in *red*, bound to 1 mm cGMP in *green*, and bound to 1 mm cCMP in *cyan*.

We also measured separations between other residues in the apo and cyclic nucleotide bound states (Figs. 6 and supplemental Fig. S3). No cyclic nucleotide-dependent changes in separation were observed for mutants with one spin label attached to residue 608 in the B-helix and another label attached to the β-roll (*top row*, Fig. 6). For mutants spin labeled at residue 616, near the B- and C-helix hinge, and the β-roll, cGMP and cCMP binding had an effect intermediate to that of cAMP (*center row*, Fig. 6). At residue 635, located at the C-terminal end of the C-helix, cAMP binding led to shifts in the distance distributions that differed from those observed in the cGMP and cCMP bound distributions (*bottom row*, Fig. 6). These data indicate that conformational changes associated with ligand binding vary in nature and extent depending on the cyclic nucleotide bound and the location in the CNBD.

##### Energetic Effects of Cyclic Nucleotide Binding to the Isolated CNBD and Intact Channel

To determine how different cyclic nucleotide-induced conformational changes in the CNBD can produce similar regulation of opening in the intact channel, we examined the energetics associated with ligand binding to the CNBD. Upon binding cAMP, the DEER measurements on residue 624 show a clear shift toward shorter separations, indicating a conformational change of the CNBD from a resting state to an activated state. However, in the ligand-free apo state, some probability density is observed at shorter separations, presumably corresponding to protein in the activated conformation (Fig. 5, *B* and *C*). Similarly, in the presence of saturating cAMP (bound state), there is still density near the position of the apo peak, indicating a fraction of ligand-bound protein is in the resting state and has not undergone a conformational change to the activated state even though ligand is bound (Fig. 5, *B* and *C*). These long, “resting” and short, “activated” distance distribution peaks can be clearly resolved and indicate an equilibrium between the resting and activated conformations that is stabilized by ligand binding. By measuring the area under these peaks, we can estimate the equilibrium constant, *L*, for undergoing the activation conformational change,

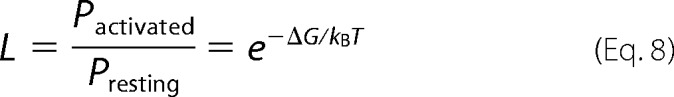
 where *P*_activated_ is the fraction of the population with distances shorter than the minimum (≈30 Å) between the apo and cyclic nucleotide bound modes and *P*_resting_ is the fraction of the population with longer distances. *L* can also be expressed in terms of Δ*G*, the standard molar free energy difference between the resting and activated states, the Boltzmann constant, *k*_B_, and the temperature, *T*. The free energy by which cyclic nucleotide binding stabilizes the activated conformation relative to the resting conformation is given by,

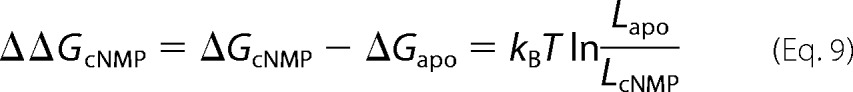
 Although Δ*G* is expected to differ between the intact channel and the isolated CNBD, ΔΔ*G* would be the same if the conformational change in the isolated CNBD is the same as in the intact channel. We estimated ΔΔ*G* for a single CNBD by integrating the peaks for the 537/624 and 563/624 mutants (Fig. 5, *B* and *C*). We measured the area under the distance distribution and under the upper and lower 2σ error bands to assess the uncertainty in area. We find ΔΔ*G*_cAMP_ ≈ −3*k*_B_*T*, ΔΔ*G*_cGMP_ ≈ −1.8*k*_B_*T*, and ΔΔ*G*_cCMP_ ≈ −2.5*k*_B_*T* with an estimated uncertainty of ≈−0.2*k*_B_*T* for these values. All ΔΔ*G* values are negative, indicating that all three cNMPs shift the equilibrium toward the activated state.

In the intact channel at saturating cyclic nucleotide concentrations, each of the four subunits will be ligand-bound. If it is assumed that the decrease in separation of residue 624 and the β-roll is entirely coupled to channel opening and that opening of the channel involves a single concerted conformational change in all four subunits, the amount by which cyclic nucleotide binding stabilizes the open state of the intact channel relative to the closed one would be four times the calculated ΔΔ*G* values, hence ΔΔ*G*_intact_ ≈ −12*k*_B_*T* for cAMP, −7*k*_B_*T* for cGMP, and −10*k*_B_*T* for cCMP.

**FIGURE 7.**
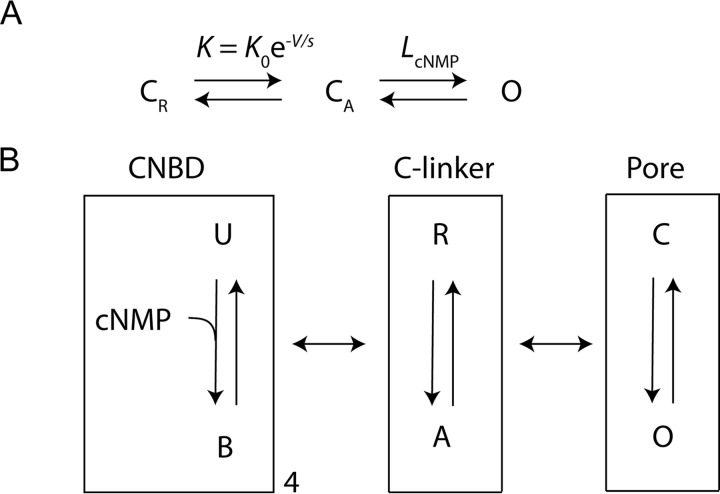
**Models for HCN2 gating.** *A*, three-state model for HCN2 gating with separate voltage-dependent activation and opening transitions. The channel can exist in a closed resting state (C_R_), a closed activated state (C_A_), and an open state (O). *K* is the equilibrium constant for voltage activation, *L* is the equilibrium constant for pore opening. *B*, modular gating scheme for HCN2. The three modules correspond to equilibria between two states each. The CNBD can be unbound (*U*) or bound (*B*) to a cyclic nucleotide ligand, the C-linker can be in a resting (*R*) or activated (*A*) state, and the pore can be closed (*C*) or open (*O*). The *double-head horizontal arrows* represent the coupling between the modules.

We can also estimate ΔΔ*G*_intact_ for the intact channel from our electrophysiology data using a simple three-state model shown in Fig. 7*A*. This model assumes two sequential transitions, a voltage-dependent transition (associated with the movement of the voltage sensor) and a voltage-independent opening transition that is affected by the binding of cyclic nucleotide. The equilibrium constant for activation of the voltage sensor is *K* = *K*_0_e^−*V/s*^ and depends on the applied voltage *V* and the slope factor, *s. K*, and therefore *K*_0_ and *s*, are assumed independent of the presence and the identity of a cNMP agonist. The equilibrium constant for channel opening, *L*, depends on the cyclic nucleotide species that is bound. The open probability *P*_o_ is given by the following equation.

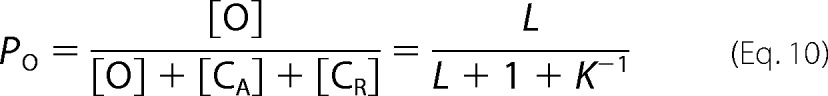


We applied this model to fit the conductance-voltage curves in the absence of cyclic nucleotide and in the presence of cAMP, cGMP, and cCMP from Fig. 1*D*. To convert the conductance to *P*_O_, we assumed an open probability of 1 for channels in the presence of cAMP and fully hyperpolarized voltages (36). The fit of the 4 dataset involves 5 free parameters: *K*_0_ and 4 *L* values. We set *s* = 3.9 mV, based on the Boltzmann fits to the conductance-voltage curves in Fig. 1*D* and fit the model in Equation 3 to the normalized conductance-voltage relationships (supplemental Fig. S4, Table 2). The fits indicated *L*_apo_ = 3.1 ± 0.5, *L*_cAMP_ = 480 ± 60, *L*_cGMP_ =260 ± 10, *L*_cCMP_ = 180 ± 50, and *K*_0_ = 7.8 × 10^−16^ ± 3.3 × 10^−16^ (values given as mean ± S.E.).

**TABLE 2:** **Three-state model parameters** Parameters for the three-state model shown in Fig. 7*A*. *K*_0_ = 7.8 × 10^−16^ ± 3.3 × 10^−16^.

Ligand	*L*	ΔΔ*G*
Apo	3.1 ± 0.5	
cAMP	480 ± 60	−5.0 *k*_B_*T*
cGMP	260 ± 10	−4.4 *k*_B_*T*
cCMP	180 ± 50	−4.1 *k*_B_*T*

From the fitted *L* values, we calculated the stabilization of the open state for the ligand bound conformation relative to the apo conformation using Equation 2. We find ΔΔ*G*_intact_ ≈ −5.0*k*_B_*T* for cAMP, −4.4.0*k*_B_*T* for cGMP, and −4.1*k*_B_*T* for cCMP. The ΔΔ*G*_intact_ estimate for cAMP is in close agreement with previous estimates for HCN2 channels based on different models and a model-independent estimate (16, 37–39). All estimates for ΔΔ*G*_intact_ are significantly smaller than the ΔΔ*G*_intact_ of −12*k*_B_*T* for cAMP suggested from our analysis of the changes in the conformational equilibrium of the isolated CNBD. Furthermore, the cyclic nucleotide potency is different, with cAMP ≥ cGMP ≥ cCMP for the intact channel and cAMP ≥ cCMP ≫ cGMP for the isolated CNBD. These results indicate that the energetic effects of binding different cyclic nucleotides to the isolated CNBD are different from their energetic effects on pore opening, suggesting the conformational changes in the CNBD are not directly or fully coupled to pore opening.

## Discussion

The binding of cyclic nucleotide ligands regulates HCN2 ion channel gating (3). In this study cAMP, cGMP, and cCMP all acted as effective agonists to increase the rate and extent of channel activation and shift the half-activation voltage toward less hyperpolarized voltages. To investigate the allosteric effect of different ligands on the HCN2 CNBD, we used NMR and DEER. Binding of different ligands produced pronounced differences in the ^1^H,^15^N-TROSY spectrum of the CNBD, indicating ligand-specific changes in chemical environments of the protein. DEER measurements on the B- and C-helices indicated that the conformations sampled by the CNBD are similar for cAMP, cGMP, and cCMP bound protein, but that the equilibrium between different conformations varies across ligands, and the ligand specificity differs from the intact channel. These results suggest that the allosteric mechanism in the CNBD is different for each cyclic nucleotide species. Despite stabilizing different conformational changes in the CNBD, the binding of the various cyclic nucleotides results in a similar effect on pore opening.

Our electrophysiology results replicated previous results for cAMP and cGMP, but found cCMP to be a more potent agonist than previously reported, a difference likely due to the expression systems or recording conditions used in our experiments. We found cCMP to be as potent of a channel activator as cGMP, and both cCMP and cGMP to be nearly as potent as cAMP. Our NMR and DEER results indicate that binding of the three cyclic nucleotides induces distinct conformational changes in the CNBD. The magnitudes of the differences in the bound conformations, however, are not revealed by the NMR data and may be small. Indeed, the x-ray crystal structures of the HCN2 C-linker/CNBD with cAMP and cGMP bound have an α-carbon root mean square deviation of only 0.6 Å (9). Furthermore, our ^1^H,^15^N-TROSY spectra indicate that neither cCMP nor cGMP have an effect that is intermediate to cAMP. This differs from a previous NMR study of a different HCN isoform, HCN4, which concluded that cCMP produced chemical shift perturbations intermediate between the cAMP and apo spectra (32).

There are at least three possible reasons for the discrepancy we observed between ΔΔ*G*_intact_ of the intact channel and ΔΔ*G* of the isolated CNBD. One is that the channel may not be activated identically by binding to each of the four identical binding sites. Indeed, previous electrophysiological studies using HCN2 channels where cAMP was prevented from binding to one or more of the subunits showed that cAMP binding does not have an identical effect for each of the four binding sites (40). Recently, by simultaneously measuring binding and gating, Kusch *et al.* (39) suggested that only the first and third binding events produce a change in the closed-open equilibrium. The mechanism for this break in symmetry for partially ligand-bound channels is unclear. However, the x-ray crystal structures of the C-linker/CNBD of HCN2 bound to cAMP or cGMP are 4-fold symmetric (9). Because the electrophysiology studies of the intact channels in this paper involve only unbound and fully bound channels, we do not think that symmetry breaking is sufficient to explain our findings.

A second possible reason for the discrepancy is that not all of the energy of the conformational change we observed in the isolated CNBD may be coupled to channel opening. Although there is good evidence in intact HCN and CNG channels that movement of the C-helix is coupled to channel opening, it could be that ΔΔ*G* from the isolated CNBD contains a component that is not directly coupled to channel opening (6, 16, 29, 41–45). This component would have to be different for cAMP, cGMP, and cCMP to account for the differences in specificity in the intact channel and the isolated CNBD. Consistent with this idea, the ΔΔ*G*s and the cyclic nucleotide specificity of the CNBD are different for DEER measurements of the rearrangements in the C-helix and B-helix. More generally, the CNBD may undergo a cyclic nucleotide-specific rearrangement that is only partially coupled to channel opening.

A third possible origin of the discrepancy is that the CNBD transition may not be directly coupled to pore opening. A possible two-step modular gating scheme was previously proposed by Craven and Zagotta (12) to suggest one way this could occur based on the structure. This scheme contains three gating modules, the CNBD, the C-linker, and the pore. The independent binding of cyclic nucleotide to the CNBDs is coupled to a concerted conformational change in the C-linker, and the conformational change in the C-linker is coupled to opening of the channel pore (Fig. 7*B*). In this model, the CNBD can be unbound (U) or bound (B), the C-linker can be in a resting (R) or activated (A) state, and the pore can be closed (C) or open (O). In the context of this Craven-Zagotta model (12), our energetic analysis of the CNBD suggests that the binding of each of the three cyclic nucleotides to the CNBD (U to B) has an almost saturating effect on the conformational transition for C-linker activation (R to A). In this model, we expect the CNBD conformation and parameters such as binding affinity and coupling between CNBD and C-linker will differ somewhat between cyclic nucleotides, but that a similar gating scheme in which binding saturates rearrangement in the C-linker applies to all cyclic nucleotides. Such saturation would explain how all three ligands can produce approximately the same effect on pore opening, but have different ΔΔ*G* values for the conformational transition in the isolated CNBD. This model is also consistent with previous studies of the C-linker. It has previously been established that the C-linker undergoes a significant rearrangement associated with cyclic nucleotide regulation of both CNG and HCN channels (11–16). These properties suggest that the C-linker is the main agent of conformational change in the intermediate module in this gating scheme (11, 12). Thus saturation of the conformational transition in the C-linker by cyclic nucleotide binding could account for the similarity of cAMP, cCMP and cGMP for pore opening despite differences in ΔΔ*G* values and CNBD conformations when cyclic nucleotide binds.

Through combining electrophysiology and magnetic resonance data, we found that, although all three nucleotides stabilize an activated conformation of HCN2, the conformational changes and degrees of stabilization of the CNBD differ. These data, combined with previous studies on the role of the C-linker, support a model where differential allosteric mechanisms in the CNBD all converge to have the same effect on the C-linker and make all three cyclic nucleotides similarly potent activators of the channel.

## Author Contributions

H. A. D., P. S. B., G. E. F., W. N. Z., and S. S. designed and analyzed the experiments. H. A. D., P. S. B., and G. E. F. carried out the experiments. H. A. D., W. N. Z., and S. S. wrote the manuscript. All authors reviewed the results and approved the final version of the manuscript.

## Footnotes

4 The abbreviations used are:

HCN: hyperpolarization-activated cyclic nucleotide-gated
CNBD: cyclic nucleotide-binding domain
DEER: double electron-electron resonance.
